# A Rare Cause of Pulmonary Nodules Diagnosed as Angiosarcoma Was Misdiagnosed as Vasculitis and Wegener’s Granuloma in an Elderly Man: A Case Report

**DOI:** 10.3389/fonc.2021.629597

**Published:** 2021-05-05

**Authors:** Peixia Wang, Liqian Xu, Yunmei Yang

**Affiliations:** Department of Geriatrics, The First Affiliated Hospital, Zhejiang University School of Medicine, Hangzhou, China

**Keywords:** Angiosarcoma, pulmonary nodule, diagnostic errors, Wegener’s granuloma, vasculitis

## Abstract

**Background:**

Angiosarcoma is a rare, highly malignant tumor prone to recurrence and metastasis. Angiosarcoma is insidious in the initial stage, and its clinical manifestation lacks specificity. The diagnosis is based on histopathology and immunohistochemistry findings.

**Case presentation:**

A 73-year-old man was hospitalized following complaints of persistent cough 6 months and hemoptysis for 2 months. Anti-infective treatment was ineffective. A CT-guided percutaneous core needle biopsy of pulmonary lesions revealed organized pneumonia, and the removed skin of purpuric rash area on the left calf revealed vasculitis. Chest CT was used during the patient follow-up. Hormonal therapy combined with immunoglobulins did not lead to improvement, and there was rapid progression of the lung lesions. Subsequently, the patient underwent a surgery, the diseased tissue was separated and removed completely beside the left submandibular gland under local anaesthesia. The immunohistochemical staining indicated CD31 (+) and CD34 (+) confirming a diagnosis of metastatic angiosarcoma. The expression of PD-L1 was 70%, therefore, anlotinib and pembrolizumab treatments were initiated. The patient eventually died.

**Conclusion:**

Angiosarcoma is a malignant tumor in the clinic that lacks typical and specific signs and symptoms. The diagnosis depends on immunohistochemistry, which requires repeated biopsies of multiple sites in highly suspected cases.

## Introduction

Angiosarcoma is a rare tumor that accounts for less than 2% of all soft tissue sarcomas (1). It is highly malignant and prone to recurrence and metastasis. These tumors can occur in any part of the body and at any age (2). Angiosarcoma is insidious in the initial stage, and its clinical manifestations lack specificity (3). The common imaging manifestations of pulmonary angiosarcoma are multiple peripheral pulmonary nodules, and diagnosis is based on histopathology and immunohistochemistry (4, 5). Immunohistochemical staining analysis is positive for the expression of tumor endothelial cell markers (CD31 and CD34) (6).

Angiosarcoma has a high mortality rate and lacks typical and specific signs and symptoms, which pose as challenges for its diagnosis and treatment by clinicians. Herein, we report the rare cause of pulmonary nodules in an elderly man. The patient had a persistent cough and hemoptysis. Owing to the non-specificity of clinical features, his condition was misdiagnosed as vasculitis and Wegener’s granuloma successively. To determine the cause, he underwent a biopsy several times. Eventually, the cause was confirmed following the resection and biopsy of a neoplasm beside the left submandibular gland, whose immunohistochemical staining suggested CD31 and CD34 positivity, indicating metastatic angiosarcoma. We discuss this case including its clinical features, diagnosis, and treatment. We also discuss the lessons we learned and the challenges experienced. It can play a certain warming role in clinical work and provide evidence for clinicians and radiologists who lack knowledge about it, to diagnose this rare entity.

## Case Report

**Figure 1:**
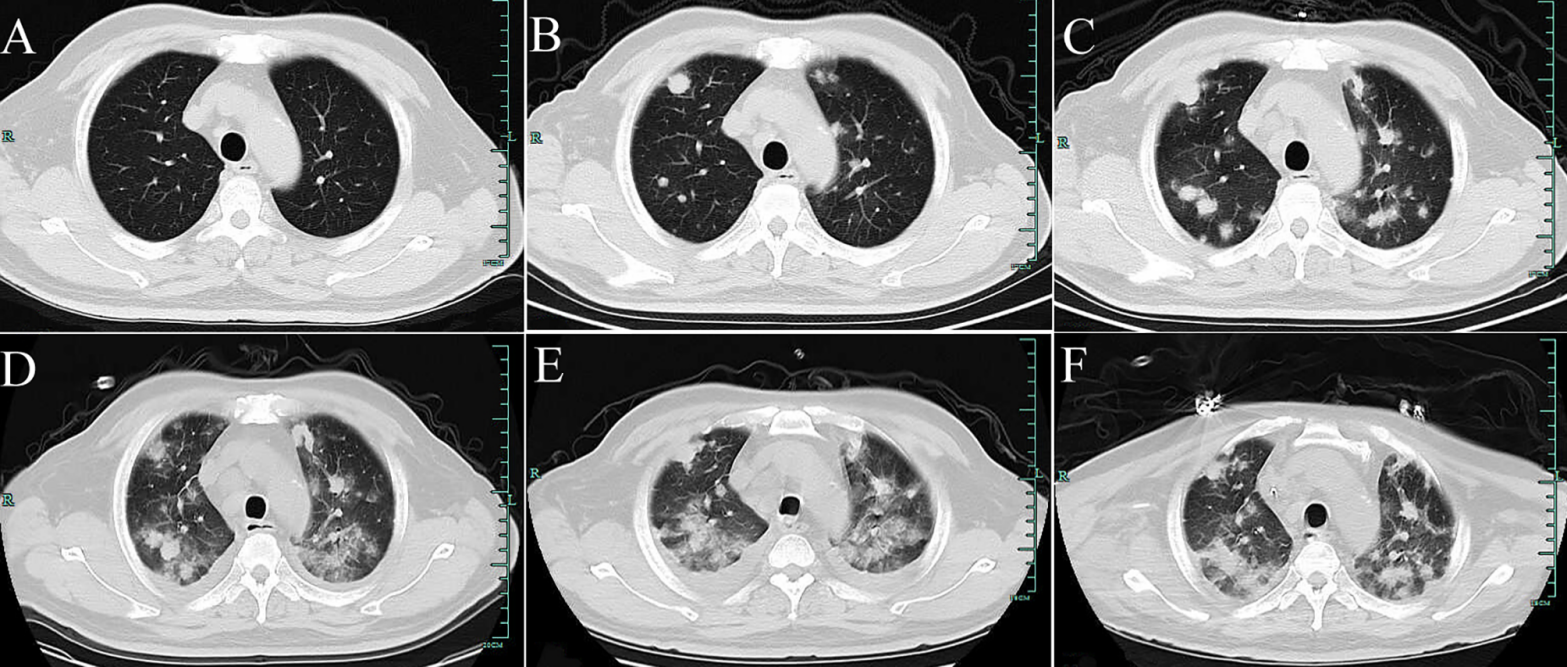
Computed tomography (CT) of the chest. **(A)** shows no positive finding in the initial imaging of both lungs on August 1, 2019. **(B)** shows scattered high-density nodular shadows of different sizes in both lungs (January 10, 2020). **(C–E)** Re-examination CT on February 17, 2020 (after antibiotics are administered); February 24 (after regular doses of hormone therapy); and February 27 (after hormone shock therapy). In both lungs, the lesions gradually progressed, demonstrating multiple scattered cotton-like, lump-like high-density shadows, and pleural effusion. **(F)** Re-examination CT after treatment with Anlotinib and Pembrolizumab for 2 weeks (March 19, 2020). The pleural effusion is decreased, and pulmonary lesions are partially resolved.

**Figure 2:**
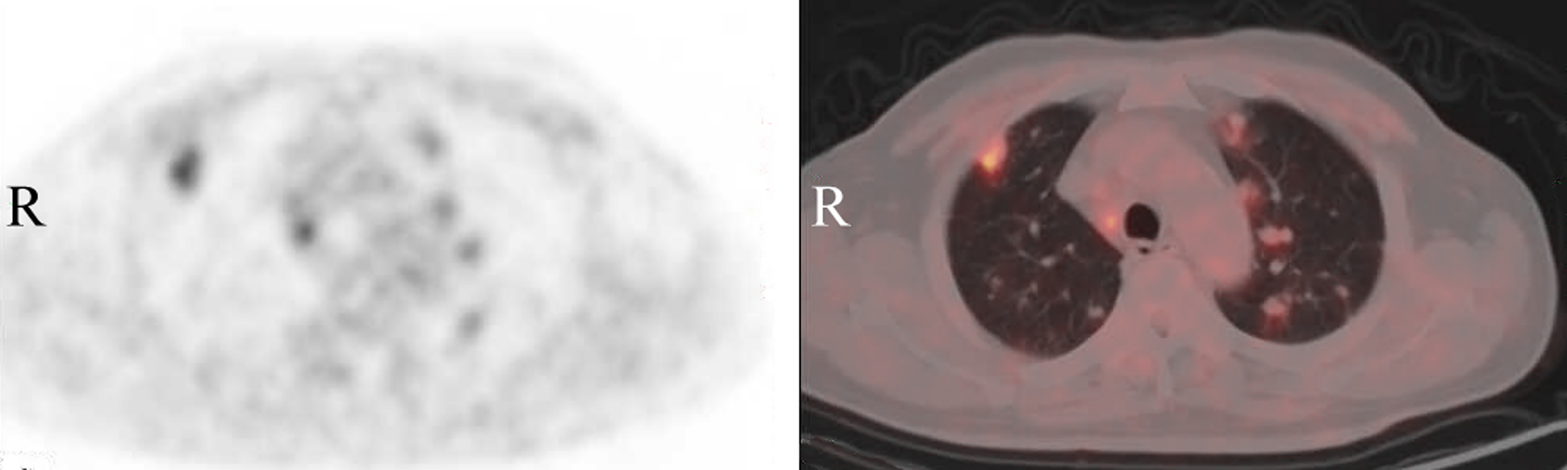
Positron emission tomography-computed tomography (PET/CT) of the chest demonstrates that multiple solid nodules of different sizes are diffused in both lungs, showing increased metabolic activity with a maximum SUV value of 4.29.

A 73-year-old nonsmoker male developed a dry cough without an obvious cause about in July, 2019. He had no other symptoms and physical examination did not reveal any significant findings. He had no personal or family history. There were no findings in the laboratory examinations and imaging examinations, Chest computed tomography (CT) scan was performed using a 64-slice spiral CT (Brilliance 64, Philips Healthcare, The Netherlands) on Aug 1, 2019 (**Figure 1A**). Cough medications were ineffective. In December 2019, dark-red blood was noticed in his sputum. There was no significant improvement with traditional Chinese medicines. Subsequently, the patient developed fever, with an elevated white blood cell count (11.0×10^9^/L, 82.6% neutrophils), C-reactive protein (CRP) of 92 mg/L, and erythrocyte sedimentation rate (ESR) of 95 mm/h. CT scan of the chest showed scattered high-density nodular shadows of different sizes with clear borders in both lungs on January 10, 2020. The diameter of the largest nodule was approximately 1.5 ×1.6 cm. Some of the nodules were surrounded by ground-glass shadow (**Figure 1B**). ^18^F-fluoro-2-deoxy-D-glucose positron emission tomography-computed tomography (^18^FDG-PET/CT) showed that multiple solid nodules of different sizes were diffused in both lungs, with a maximum standardized uptake values (SUV) of 4.29 (**Figure 2**). He was administered intravenous moxifloxacin (400 mg daily) and linezolid (600 mg every 12 h) in the clinic.

When the patient was admitted to the hospital, his temperature, respiration, blood pressure, and heart rate were normal. His chest was clear, and his abdomen was soft, with no guarding or rigidity. Purpuric rashes were scattered on both calves at 0.1–1 cm, without fading under pressure, and without breaking of the skin. His blood tests indicated a white blood cell count of 9.9 × 10^9^/L, with 87% neutrophils, and hemoglobin levels of 86 g/L. He had a slightly reduced CRP level of 69 g/L, reduced ESR of 65 mm/h, and rheumatoid factor of 33.5 IU/mL. His other laboratory data, including anti-endothelial cell antibody, anti-neutrophil cytoplasm autoantibodies, and antinuclear antibody were unremarkable. He was advised to undergo a lung biopsy, however, he refused. While he accepted a skin biopsy, the area of purpuric rash on the left calf was removed using a scalpel, which revealed vasculitis. When the sputum culture and next-generation sequencing suggested Mycobacterium chelonae, antibiotics, posaconazole (300 mg daily) and methylprednisolone (40 mg daily) were given. However, cough and blood sputum persisted. The CT was used for the patient follow-up, and the imaging revealed worse findings than the previous one, on February 17, 2020 (**Figure 1C**).

**Figure 3:**
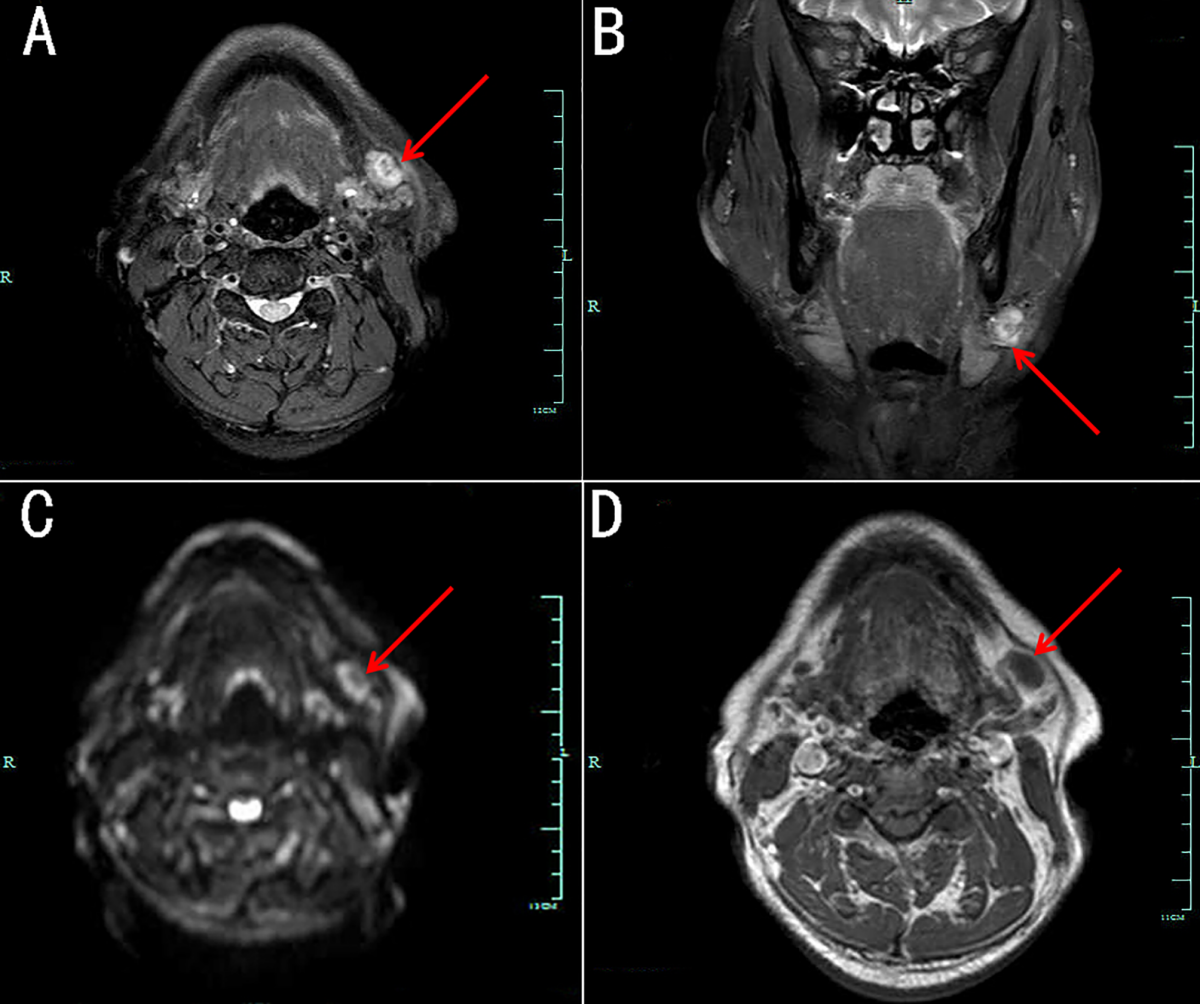
Magnetic resonance imaging (MRI) of a lesion beside the left submandibular gland. The lesion demonstrates heterogeneous hyper-intensity on axial **(A)** and coronary **(B)** fat-suppressed T2-weighted images, as well as on axial diffusion-weighted images **(C)**. Axial T1-weighted image **(D)** showing slight hypo-intensity.

He agreed to undergo a CT-guided percutaneous core needle biopsy of the nodule in the upper lobe of the right lung at 120 kVp tube voltage (Philips Healthcare, The Netherlands), and the pathology suggested organized pneumonia. Experienced physicians in the departments of respiratory diseases, hematology, rheumatology, infectious diseases and radiology participated in multidisciplinary discussions, and systemic vasculitis was first considered for which hormone therapy (methylprednisolone 80 mg every 12h) was recommended. During the treatment period, a soft, mobile, non-tender mass was detected beside his left submandibular gland, with the diameter of 1.7 ×1.3 cm using ultrasound scan (Logic 9, GE Healthcare, Horton, Norway). When magnetic resonance imaging (MRI) scan was performed using a 3.0 T scanner (Achieva, Philips Healthcare, The Netherlands) with a neck matrix coil, the lesion demonstrated heterogeneous hyper-intensity on axial and coronary fat-suppressed T2-weighted images, as well as on the axial diffusion weighted image. Axial T1-weighted image showed slight hypo-intensity around the left submandibular gland (**Figure 3**). It was considered a benign lesion based on a stomatology consultation.

Since hemoptysis did not relieved obviously, the patient was advised to undergo a biopsy again. A bronchoscopic lung biopsy was performed using endobronchial ultrasound-guide sheath, which pathology suggested chronic inflammation. However, he had a severe hemoptysis. Re-examination by CT scan showed that there was an obvious halo sign around the focus, which indicated a lesion combined with bleeding (**Figure 1D**) and pulmonary auscultation showed bilateral rales. Although antibiotics and hormone therapy were administered for more than a month, the lung lesions still progressed. Multiple experts from our hospital and Shanghai Ruijin Hospital, including those from departments of respiratory diseases, rheumatology, infectious diseases, radiology, and pathology after repeated discussions, concluded on February 24, 2020 that Wegener’s granuloma could not be excluded. Therefore, methylprednisolone 1000 mg was administered intravenously every day for 3 days with other treatments including immunoglobulin and thymosin to boost the immune system of the patient. Owing to the deterioration in symptoms and CT imaging (**Figure 1E**), experts of the above departments from Peking Union Medical College Hospital also participated in the multidisciplinary discuss of the patient online. They concluded that angiosarcoma should be considered as the cause of hemoptysis, and a thoracoscopic lung biopsy was proposed for the lung lesion. However, the hemoptysis worsened, accompanied by aggravated chest tightness and oxygen saturation of 90% (SpO_2_), and the patient could not tolerate the surgery.

**Figure 4:**
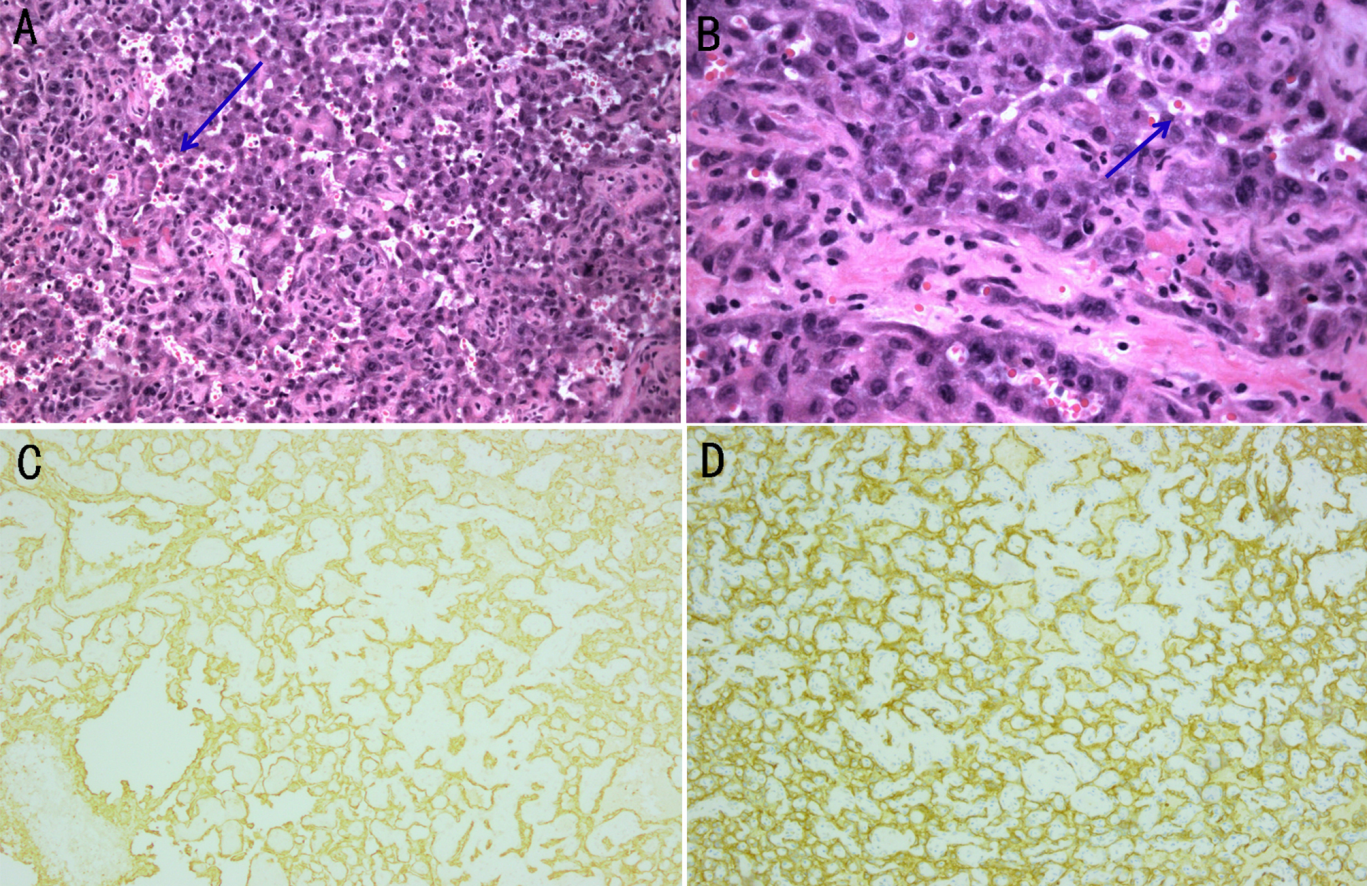
Histological examination and immunohistochemical analysis of the biopsy specimen beside the left submandibular gland. **(A, B)** The gland is composed of irregular dilated vascular lacunae, which are filled with red cells. Vascular channel-like structure with vague lumen formats by abnormal, pleomorphic, malignant endothelial cells, which can be rounded, polygonal, or fusiform and can have an epithelioid appearance. The arrows point to the red cells. Magnification of **(A)** is 100×, and **(B)** is 200×. **(C)** The expression of CD31 is determined using immunohistochemical staining (100× magnification). **(D)** The expression of CD34 is determined using immunohistochemical staining (100× magnification).

After multidisciplinary discussions, his family members agreed to a repeated biopsy to clarify the cause. When the patient’s respiratory failure improved, following the opinions of experts in respiratory diseases, radiology, oncology, thoracic surgery and pathology departments, the surgeon separated the neoplasm and removed it completely beside the left submandibular gland under local anesthesia. The histopathology of surgical tissue sections showed that it was a metastatic tumor, and immunohistochemical analysis revealed CD31 and CD34 expression (**Figure 4**), which confirmed the diagnosis of metastatic angiosarcoma. Thus, the cause of hemoptysis and pulmonary nodules was finally identified as angiosarcoma. Anlotinib (12 mg every day) was administered, and next-generation sequencing was performed, which showed that no effective targeted drugs. The expression of PD-L1 was 70%. Pembrolizumab 200 mg (an immune checkpoint inhibitor that targets the PD-1 receptor of lymphocytes) was administered intravenously. After 2 weeks of treatment, the pulmonary lesions showed that pleural effusion decreased, and pulmonary lesions partially resolved (**Figure 1F**). However, hemoptysis did not improve significantly and respiratory failure was detected. Various treatments did not terminate the disease progression; with the gradual development of multiple serosal cavity effusions, liver failure, heart failure, and disseminated intravascular coagulation, the patient eventually died less than a month after diagnosis.

## Discussion

### Clinical Features

Angiosarcoma was first reported by Caro in 1945. It is a highly malignant tumor originating from vascular or lymphatic endothelial cells. It was previously referred to as hemangioendothelioma, malignant hemangioendothelioma, adenosarcoma, angiosarcoma and lymphangiosarcoma and is now collectively referred to as angiosarcoma (7). Angiosarcoma most commonly occurs in the skin of the head and neck, particularly on the scalp, in elderly individuals, accounting for approximately half of all of these tumors (3). Other common sites include the breast, thyroid, heart, liver, spleen, pulmonary vessels and limbs. The degree of malignancy is extremely high, and it mainly spreads through the blood, which makes it prone to recur and metastasize (8). The lung is the most common site of metastasis (9).

Angiosarcoma manifests insidiously at the initial stage and lacks specific manifestations; up to 20% of patients are asymptomatic, and the disease is accidentally discovered during autopsy (10). Pulmonary angiosarcoma can cause chest pain, chest tightness, hemoptysis, dyspnea, cough and other symptoms; a large number of patients have cough and hemoptysis as the first diagnostic symptoms (11).

Clinical symptoms can provide clues for diagnosis, however, imaging examinations are also essential for definitive diagnosis. Angiosarcoma shows multiple solid nodular lesions in lung CT scan, which could be accompanied by marginal ground-glass shadows or ground-glass nodules, along with multiple thin-walled cysts (12, 13). Most solid lesions show non-homogeneous enhancement. These imaging findings, whether solid or cystic, can generally be observed with hemorrhagic changes, which are considered to be a characteristic finding of metastatic angiosarcoma. Radiologically, these hemorrhagic complications may be characterized by gas-fluid levels in thin-walled cysts, diffuse pulmonary infiltration, hemothorax or ground-glass thinning areas (11, 14, 15). PET/CT can non-invasively distinguish benign and malignant lesions, search for metastatic lesions, and provide clinical decision-making ideas for diagnosis by combining patients’ clinical symptoms and related examinations, which has a high negative predictive value (16). The main manifestation of angiosarcoma on PET/CT was increased metabolism of ^18^F-FDG, with an SUV value between 6.7 and 22 (17, 18).

### Diagnosis and Treatment

A biopsy is essential for the diagnosis of angiosarcoma. The diagnosis of angiosarcoma is based on histological examination and immunohistochemical analysis. Histological examination revealed the hallmarks of angiosarcoma, such as abnormal, pleomorphic, and malignant endothelial cells, which can be rounded, polygonal, or fusiform and may have an epithelioid appearance (10) and vascular channel-like structure with vague lumen formats by these cells. Immunohistochemically, the neoplastic cells are positive for ERG, CD31 and CD34. CD31 can be detected in approximately 90% of the cases, which is relatively specific and extremely sensitive, particularly in poorly differentiated cases (19, 20). Owing to the rarity of angiosarcoma, there is no standardized treatment regimen established. Angiosarcoma can be treated with radiotherapy, surgical resection, immunotherapy, chemotherapy, or a combination of these in cases with localized disease as reported (20–22). Surgery is considered to be the most effective treatment. However, most of the neoplasms lose the opportunity of operation at the time of diagnosis. Multimodality therapy has proved to be effective (21).

The pathogenesis of the rare disease has not yet been fully elucidated. Since angiosarcoma originates from blood vessels or lymphatic vessels, the abnormal activation of vascular endothelial growth factor (VEGF) and its receptor (VEGFR) may be responsible for angiosarcoma formation. Recent studies have shown that VEGF and VEGFR play an important role in tumor angiogenesis, and the overexpression of VEGF is observed in 21%–25% of all patients with soft tissue sarcoma (23). Targeting VEGF-related proteins has become the focus of research for treating angiosarcoma (24, 25). Anlotinib, an oral multi-targeted tyrosine kinase receptor inhibitor that can inhibit the formation of new blood vessels, inhibiting downstream signal transduction mediated by VEGFR2 (26).

The fatality rate is quite high, the overall 5-year survival rate of angiosarcoma is approximately 35%. Angiosarcoma of the skin is easy to detect, and when it does not involve the main organs, the prognosis is better than that of other parts. According to current literature reports, individuals aged <70 years old, with a tumor diameter <5 cm, located on the trunk have a good prognosis, and approximately 60% of these individuals survive for more than 5 years. Highly aggressive angiosarcomas with a median survival time of the disease of 7 months, have an overall poor prognosis (5). The mortality of pulmonary angiosarcoma within the first few months after clinical presentation approaches 100%, most patients die within a few months after the first visit. Due to its rarity and challenges during treatment, clinical studies with large sample sizes to establish standardized guidelines for diagnosis and treatment are difficult to conduct (23, 27). Timely diagnosis and early treatment are key to effectively prolonging the survival time of patients.

### Findings in This Case

While reviewing the literature, we discovered that our patient had a few different and some similar characteristics. We report a rare cause of pulmonary nodules: angiosarcoma. The patient had persistent cough and hemoptysis. The clinical manifestations were highly similar to pulmonary thromboembolism, vascular malformations, lung cancer, severe pneumonia, and tuberculosis; thus, misdiagnosis was easy. The initial symptoms of the patient were consistent with those reported in the literature (13, 28), therefore, posing diagnostic challenges for clinicians.

During the imaging examination, the CT scan in this patient showed multiple solid pulmonary nodules with a ground-glass shadow initially and hemorrhagic changes and pleural effusion in the later stage as the disease progressed. FDG-uptake on PET/CT scans was lower than that of general malignant tumors. The atypical imaging changes of malignant tumors, which was not consistent with the previous imaging findings of angiosarcoma, indicated that the biological characteristics of angiosarcoma were different from those of other malignant tumors, thus, enhancing the challenges of clinical diagnosis. Moreover, repeated pathological examination and biopsy of different sites showed no sign of a malignant tumor, these were conducive to the occurrence of misdiagnosis or diagnostic error. Eventually diagnosis of angiosarcoma using biopsy, histology and immunohistochemistry were both consistent with angiosarcoma, as reported in the literature (13, 14).

Presently, there are no obvious effective treatments, and the disease progresses rapidly. The patient was treated with anlotinib as suggested by experts, there were many reports showing that the drug could treat several types of malignant tumors (24–26). Pembrolizumab may contribute to the antitumor effect (25). However, the patient died approximately 5 months after diagnosis, which was most consistent with other cases reported in the literature.

### Lessons and Limitations

Reviewing the experience in this case, despite multiple clinical findings pointing toward a malignant entity, the negative results of two lung biopsies and non-specificity of the clinical features and the atypical imaging changes of malignant tumors, lead to a delay in the diagnosis of the cause. The following lessons can be learned. First, symptoms were cough and hemoptysis, accompanied by fever and an increased inflammation index, which is a characteristic of infectious diseases. Moreover, most of lesions in CT imaging are prone to inflammatory changes. According to clinical experience, infectious disease was considered first, and the opportunity for early diagnosis was missed. Second, malignant tumors generally show hypermetabolism in PET/CT examination, which is inconsistent with the reports. This shows that PET/CT has limitations in the diagnosis of low-metabolic malignancies. Third, biopsy has a certain negative rates, it depends on the puncture method, location and the size of the lesion. In this case, the patient underwent lung biopsies, twice, and both did not indicate a malignant tumor. We assumed that the error in pathological results were owing to fewer samples obtained by puncture biopsy and various degrees of inflammation around the lesions probably, which might have affected the correct diagnosis (28, 29). Therefore, tissue samples could be obtained by multi-site or video-assisted thoracoscopic biopsies, which could effectively improve the positivity rate of biopsy. Moreover, the first manifestations were hemoptysis and pulmonary nodules. However, primary pulmonary angiosarcoma is reported to be extremely rare (23). In this case, the primary site was first considered as the lung, however, the possibility of lung metastasis at other primary sites was not excluded. Finally, multidisciplinary consultation plays an important role in the diagnosis of some rare diseases.

When the clinical features lack of specificity, physical examination plays an important role in the diagnostic process as it they may uncover an alternative cause of the patient’s symptoms. The clinicians must pay attention to the new signs and symptoms. While examining a case with clinical findings not matching the pathology, the pathologist should be consulted.

## Conclusion

Angiosarcoma lacks typical and specific signs and symptoms. The diagnosis depends on immunohistochemistry, which requires repeated biopsies of multiple sites in highly suspected cases. The present case provides evidence of a rare cause of pulmonary nodules and hemoptysis and play a certain warning role in the diagnosis and treatment of similar diseases. It also contributes to the existing knowledge in the subject and will aid clinicians and radiologists in diagnosing this rare entity.

## Data Availability Statement

The original contributions presented in the study are included in the article/**Supplementary Material**. Further inquiries can be directed to the corresponding author.

## Ethics Statement

Written informed consent was obtained from the individual(s) for the publication of any potentially identifiable images or data included in this article.

## Author Contributions

PW was a major contributor in writing the manuscript. LX and YY were main physicians for the patient, and proofread and revised the manuscript. All authors contributed to the article and approved the submitted version.

## Conflict of Interest

The authors declare that the research was conducted in the absence of any commercial or financial relationships that could be construed as a potential conflict of interest.
