# Supplementary material for: A Rare Cause of Pulmonary Nodules Diagnosed as Angiosarcoma Was Misdiagnosed as Vasculitis and Wegener’s Granuloma in an Elderly Man: A Case Report
Source: Front Oncol. 2021 May 5;11:629597. doi: 10.3389/fonc.2021.629597 (PMC8131857; doi:10.3389/fonc.2021.629597)
Supplement: Supplementary file 1 [file DataSheet_1.pdf]

CARE case report guidelines

CARE Checklist of information to include when writing a case report

(cc) BY-NC-ND

Topic	Item	Checklist item description	Reported on Line
Title	1	The diagnosis or intervention of primary focus followed by the words "case report"	L 3-4
Key Words	2	2 to 5 key words that identify diagnoses or interventions in this case report, including "case report"	L39
Abstract (no references)	3a	Introduction: What is unique about this case and what does it add to the scientific literature?	L14-16
	3b	Main symptoms and/or important clinical findings	L19-20
	3c	The main diagnoses, therapeutic interventions, and outcomes	L29-31
	3d	Conclusion—What is the main "take-away" lesson(s) from this case?	L35-37
Introduction	4	One or two paragraphs summarizing why this case is unique (**may include references**)	L51-61
Patient Information	5a	De-identified patient specific information.	L19，L63
	5b	Primary concerns and symptoms of the patient.	L63-64,L69-70 ,L134-135
	5c	Medical, family, and psycho-social history including relevant genetic information	L63, L65
	5d	Relevant past interventions with outcomes	L68-69，L70-71
Clinical Findings	6	Describe significant physical examination (PE) and important clinical findings.	L71-80，83-91,105-113 ,137-144
Timeline	7	Historical and current information from this episode of care organized as a timeline	L64，68，75，98
Diagnostic Assessment	8a	Diagnostic testing (such as PE, laboratory testing, imaging, surveys).	L92-93，99-101，115-116，140-145
	8b	Diagnostic challenges (such as access to testing, financial, or cultural)	L92，99-101，140-145，134-136
	8c	Diagnosis (including other diagnoses considered)	L142-145
	8d	Prognosis (such as staging in oncology) where applicable	L144
Therapeutic Intervention	9a	Types of therapeutic intervention (such as pharmacologic, surgical, preventive, self-care)	L145-146，148-149
	9b	Administration of therapeutic intervention (such as dosage, strength, duration)	L145-146，148-150
	9c	Changes in therapeutic intervention (with rationale)	L151-154
Follow-up and Outcomes	10a	Clinician and patient-assessed outcomes (if available)	L151-155
	10b	Important follow-up diagnostic and other test results	L146-147
	10c	Intervention adherence and tolerability (How was this assessed?)	L149-152
	10d	Adverse and unanticipated events	L151-152
Discussion	11a	A scientific discussion of the strengths AND limitations associated with this case report	L255-283
	11b	Discussion of the relevant medical literature **with references**.	L158-228
	11c	The scientific rationale for any conclusions (including assessment of possible causes)	L230-254
	11d	The primary "take-away" lessons of this case report (without references) in a one paragraph conclusion	L279-283
Patient Perspective	12	The patient should share their perspective in one to two paragraphs on the treatment(s) they received	L259-278
Informed Consent	13	Did the patient give informed consent? Please provide if requested	Yes ☑ No ☐
